# Resistance of Mycorrhizal *Cinnamomum camphora* Seedlings to Salt Spray Depends on K^+^ and P Uptake

**DOI:** 10.3390/jof9100964

**Published:** 2023-09-26

**Authors:** Lin Xue, Peng Liu, Aiping Wu, Lijia Dong, Qiqian Wu, Mingshui Zhao, Hua Liu, Yan Li, Naili Zhang, Yanhong Wang

**Affiliations:** 1 State Key Laboratory of Subtropical Silviculture, Zhejiang A & F University, Hangzhou 311300, China; 2021602122121@stu.zafu.edu.cn (L.X.); 2022602121078@stu.zafu.edu.cn (P.L.); qiqianwu@zafu.edu.cn (Q.W.); liuhua@zafu.edu.cn (H.L.); liyan2016@zafu.edu.cn (Y.L.); 2 Ecology Department, College of Environment and Ecology, Hunan Provincial Key Laboratory of Rural Ecosystem Health in Dongting Lake Area, Hunan Agricultural University, Changsha 410128, China; wuaiping8101@126.com; 3 School of Life and Environmental Sciences, Shaoxing University, Shaoxing 312000, China; donglijia@126.com; 4 Zhejiang Tianmu Mountain National Nature Reserve Administration, Hangzhou 311311, China; tmszms@163.com; 5 State Key Laboratory of Efficient Production of Forest Resources and the Key Laboratory of Silviculture and Conservation of Ministry of Education, Beijing Forestry University, Beijing 100083, China

**Keywords:** salt spray, arbuscular mycorrhizal fungi, root trait, salinity tolerance, mycorrhizal colonization

## Abstract

Salt spray is a major environmental issue in coastal areas. *Cinnamomum camphora* is an economically important tree species that grows in the coastal areas of southern China. Arbuscular mycorrhizal fungi (AMF) can alleviate the detrimental effects of abiotic stress on host plants. However, the mechanism by which AMF mitigates the adverse effects of salt spray on *C. camphora* remains unclear. A pot experiment was conducted in a greenhouse, where *C. camphora* seedlings were exposed to four AMF regimes (inoculation with sterilized fungi, with *Glomus tortuosum*, *Funneliformis mosseae*, either alone or in combination) and three salt spray regimes (applied with distilled water, 7, and 14 mg NaCl cm^−2^) in order to investigate the influence on root functional traits and plant growth. The results showed that higher salt spray significantly decreased the K^+^ uptake, K^+^/Na^+^ ratio, N/P ratio, total dry weight, and salinity tolerance of non-mycorrhizal plants by 37.9%, 71%, 27.4%, 12.7%, and 221.3%, respectively, when compared with control plants grown under non-salinity conditions. Mycorrhizal inoculation, particularly with a combination of *G. tortuosum* and *F. mosseae*, greatly improved the P uptake, total dry weight, and salinity tolerance of plants grown under higher salt spray conditions by 51.0%, 36.7%, and 130.9%, respectively, when compared with their counterparts. The results show that AMF can alleviate the detrimental effects of salt spray on *C. camphora* seedlings. Moreover, an enhanced uptake of K^+^ and P accounted for the resistance of the plants to salt spray. Therefore, pre-inoculation with a combination of *G. tortuosum* and *F. mosseae* to improve nutrient acquisition is a potential method of protecting *C. camphora* plants against salt spray stress in coastal areas.

## 1. Introduction

*Cinnamomum camphora* (L.) Presl, a camphor tree, is widely distributed along the Yangtze River Basin in southern China, as well as in Southeast and East Asia [1]. The species is economically important and has historically played crucial roles in protecting coastal areas against erosion, owing to its highly developed root system, rapid growth, and longevity [2,3]. In China, the total length of the coastline stretches up to 3.2 × 10^4^ km, with over 50% of major cities and 60% of the gross domestic product concentrated in coastal areas [4]. To successfully cultivate *C. camphora* in such a challenging habitat, one must mitigate the undesirable effects of salt spray, a natural stress factor intolerable to many plant species [4,5].

The annual global salt spray emissions range from 0.02 to 1 × 10^14^ kg per year [6]. Salt spray comprises fine salt droplets that can penetrate the aerial parts of plants and redistribute in the soil after precipitation under the combined effects of gravity and wind [7,8]. The primary component of salt spray is NaCl [9], with intensity at a plant height of 1 m ranging from 1 to 200 mg NaCl dm^−2^ d^−1^ [8,10]. Previous studies have demonstrated that salt spray can have either positive or negative effects on plant growth and development, depending on wind speed, distance from seawater, site topography, and plant characteristics [11,12,13,14,15]. In recent years, the effects of salt spray on plant growth have been exacerbated due to the increased frequency of heavy storms and the extension of coastal erosion [5]. Notably, salt spray adversely affects plant growth by disrupting water balance [16,17], reducing photosynthetic capabilities [18,19], and inhibiting nutrient uptake [3,20], occasionally resulting in plant mortality [5]. In particular, the decline in nutrient acquisition is reportedly the primary cause of the adverse effects of salt spray on plant growth [3]. Therefore, enhancing nutrient absorption may be preferable for plants grown in coastal areas. Damage to plants by salt spray is well documented, mainly in terms of growth inhibition [10]. However, few studies have explored biological methods for improving resistance to salt spray [5,20]. Hence, approaches that facilitate increased nutrient uptake by plants would be helpful in improving the ability to withstand salt spray.

Among the suggested biotechnological approaches, the interaction between plants and arbuscular mycorrhizal fungi (AMF), the most widespread symbiotic relationship in nature, is of great importance in stressful conditions [21]. In this beneficial interaction, the fungus receives photosynthetic carbon from colonized plants and reciprocally provides their host plants with water and nutrients, particularly phosphorus (P) and nitrogen (N) [22]. Compelling evidence indicates that AMF enhances the stress tolerance of host plants [23,24,25,26,27]. Several well-known mechanisms contribute to mycorrhizal efficiencies, such as nutrient uptake improvement, ionic homeostasis maintenance, photosynthetic capabilities enhancement, antioxidant metabolism reinforcement, cell ultrastructure preservation, and root system architecture alteration [28,29,30,31]. Mycorrhizal effects can range from positive to parasitic, depending on specific plant–AMF combinations and environmental conditions [30,32]. 

Notably, *C. camphora* poorly adapts to soil salinity [33,34,35] and is mycotrophic, with root colonization reaching up to 81.85% [36]. However, the protective effects of AMF on salt-sprayed *C. camphora* seedlings, and the underlying mechanisms, remain unclear, which is the new aspect of this research compared to previous studies. Considering that mycorrhizal symbiosis can strengthen nutrient uptake [30,37], we hypothesized that AMF inoculation mitigates the adverse effects of salt spray on *C. camphora*. To test this hypothesis, seedlings of *C. camphora* were experimentally pretreated with AMF inoculation and simulated salt spray was applied on the aerial parts, and consequently, their response patterns in terms of plant growth, root morphological traits, nutrient uptake, and ionic ratios were determined. The results of this study provide insights into the mechanism underlying the mycorrhiza-mediated alleviation of salt spray stress as well as guidance on growing *C. camphora* plants in coastal areas.

## 2. Materials and Methods

### 2.1. Plant Materials and Experimental Design

*C. camphora* seedlings were grown from seeds obtained in December 2020 at the research station of Zhejiang A & F University (30°14′ N, 119°42′ E) near Hangzhou Bay, East Sea of southeastern China [38]. The seeds were surface-sterilized (70% ethanol for 1 min, 2.625% NaClO for 3 min, 70% ethanol for 1 min, and distilled water for 1 min) [39] and maintained at 4 °C until February 2021. On 22 February 2021, the seeds were sown in autoclaved sand for germination [40]. Two months later, 60 germinated seedlings at the same growth stage (~4 cm in height and three leaves per plant) were selected and transplanted into plastic pots (16.5 cm × 18 cm × 12 cm; one seedling per pot). Each pot was filled with 2 kg of local soil collected from the topsoil (0–10 cm) at the research station, thoroughly mixed, and sterilized with gamma irradiation at 25 kGy [41]. The soil had a pH of 5.83 (soil-water ratio: 1:5) and contained 22.31 mg·g^−1^ organic matter, 0.083 mg·g^−1^ available nitrogen, 0.28 mg·g^−1^ Olson P, 2.42 mg·g^−1^ potassium (K^+^), 0.16 mg·g^−1^ sodium (Na^+^), 1.21 mg·g^−1^ calcium (Ca^2+^), 1.62 mg·g^−1^ magnesium (Mg^2+^), 0.008 mg·g^−1^ copper (Cu^2+^), 0.048 mg·g^−1^ zinc (Zn^2+^), 14.77 mg·g^−1^ iron (Fe^2+^), and 0.297 mg·g^−1^ manganese (Mn^2+^). During the experimental period, the plants were grown in a naturally lit greenhouse with a midday photosynthetic photon flux density of 1200 μmol m^−2^ s^−1^, an average temperature of 29 °C, and a relative humidity of 70%. All pots were rearranged at regular intervals to minimize the influence of environmental factors.

The experiment was conducted using a factorial design involving twelve treatment combinations composed of four AMF and three salt spray regimes. Each treatment combination was replicated five times. For the AMF treatments, two AMF that are prevalent in stressful environmental conditions were selected as mycorrhizal inocula: *Glomus tortuosum* N. C. Schenck and G. S. Smith (BGC NM05A) [42] and *Funneliformis mosseae* (T. H. Nicolson and Gerd) C. Walker and A. Schüßler (BGC XJ02) [37]. Both were supplied by the Bank of Glomeromycota of China (BGC) (Beijing, China) and consisted of hyphae, spores, and colonized root fragments of sorghum (*Sorghum bicolor* L.) [38]. When transplanting, mycorrhizal plants (AM plants) were provided with 40 g of a single AM fungus (*G. tortuosum* or *F. mosseae*) or 40 g of combined inoculum composed of equal ratios of the two fungi, and mycorrhizal inocula were added at a depth of 10 cm below the growth substrate. Non-mycorrhizal control plants (NM plants) received 40 g of autoclaved combined fungal inoculum and 40 mL of the filtrate of the fungal inoculum to reintroduce microflora to the sterilized soil [43]. To prevent negative effects on the development of the fine roots of plants and on AMF establishment, salt spray treatment was initiated on 22 May 2021, one month after seedling transplantation. Salt spray was applied using a handheld herbicide applicator that produced a fine mist at a concentration of 7 mg NaCl cm^−2^ (low salt spray concentration, LS) or 14 mg NaCl cm^−2^ (high salt spray concentration, HS). These values were equivalent to the accumulation rates observed in plants growing close to the ocean in southeastern China [44]. All treated seedlings were sprayed once with 100 mL of the corresponding NaCl solution between 16:00 and 18:00 pm each day. Untreated control plants received an identical application of deionized water to account for any physical effect of the spraying on the plants [8]. During the experiment, no additional fertilizer was added, and the plants were watered when needed. After six consecutive months of salt spray treatment, all seedlings were harvested on 22 November 2021.

### 2.2. Determination of Root Morphological Traits and Plant Biomass

At harvest, all plants were gently separated from the growth substrate, rinsed several times with deionized water, and then divided into shoots and roots to measure the fresh biomass. All fresh roots were placed in sampling boxes containing ice and transported to the laboratory for the determination of root surface area, volume, diameter, and total length using the WinRHIZO root analysis system (Regent Instruments, Canada). Subsequently, each root sample was divided into two subsamples, and the fresh biomass was recorded. One of the root subsamples was kept in FAA solution (formalin-aceto-alcohol) [45] to determine the root colonization by AMF. The remaining plant shoots and roots were weighed after oven-drying at 60 °C for 72 h. The dry weights of the root subsamples used for mycorrhizal colonization were calculated according to the method described by Veiga et al. [46]. The dry weight of each root sample was computed by summing the two root subsamples.

### 2.3. Determination of Root Colonization

Root colonization by fungal species was determined using a previously reported method [38]. Briefly, 0.5 g of root segments were cleared with 10% KOH at 90 °C for 4 h, acidified with 1 M HCl for 2 min, and stained with 0.05% trypan blue for 35 min [47]. The stained roots were then mounted on slides, and root colonization by AMF was microscopically examined using the gridline intersection method [48].

### 2.4. Determination of Elemental Concentrations

Plant samples (shoots or roots) from three randomly selected plants in each experimental manipulation were individually ball-milled to measure their elemental concentrations. Nitrogen (N) and phosphorus (P) concentrations were determined using a continuous flow analyzer (SEAL-AA3, Bran-Luebbe Inc., Hamburg, Germany) [49]. The plant samples were digested with 5 mL of 18 M H_2_SO_4_ for ~1 h, and 30% H_2_O_2_ was subsequently added for further digestion. After cooling, the digestion solution was diluted with deionized water and used to measure the N and P concentrations with the aforementioned analyzer. Potassium (K^+^) and sodium (Na^+^) concentrations were determined using an AA-7000 atomic absorption spectrophotometer (Shimadzu, Tokyo, Japan), following the protocol described by Chapman and Pratt [50].

### 2.5. Data Analysis

To quantify the mycorrhizal efficiency, the mycorrhizal growth response (MGR) was calculated using the following equation [51]:

(1) $$MGR\left(\%\right)={log}_{e}\left(\frac{{DW}_{AMF}}{Avg\left({DW}_{non-AMF}\right)}\right)\times 100\%,$$

where *DW_AMF_* is the total dry weight of AM plants, and *Avg*(*DW_non−AMF_*) is the mean dry weight of NM plants grown under identical salt spray conditions.

Salinity resistance (ST) was calculated using the following equation [52]:

(2) $$ST(\%)=\frac{W_{1}-W_{2}}{W_{2}}\times 100\%,$$

where *W*_1_ is the dry weight of the plant under salt spray stress, and *W*_2_ is the average dry weight of the control plants.

The data were analyzed using two-way ANOVA to assess the effects of salt spray, AMF, and their interactions. Before the analysis, all data were subjected to homogeneity and normality tests. When the interaction between salt spray and AMF was significant, Fisher’s least significant difference (LSD) test was used to determine the differences between the treatment groups. Pearson’s correlation analysis was performed to determine the relationships among all the determined parameters. Furthermore, stepwise regression models were used to quantify the contribution of the determined plant parameters to MGR and ST [53]. The aforementioned analyses were conducted using the R.4.1.1 statistical platform (http://www.R-project.org, accessed on 3 December 2022).

## 3. Results

### 3.1. Mycorrhizal Colonization

**Figure 1:**
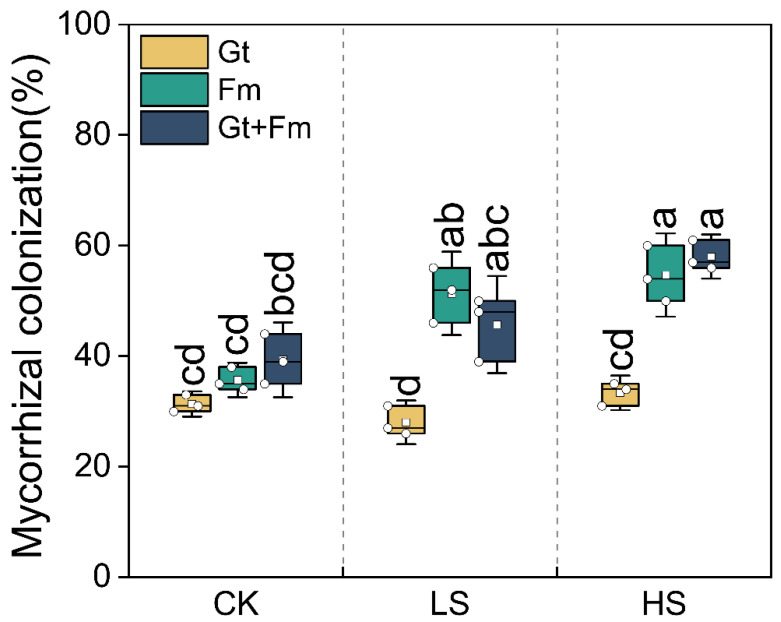
Mycorrhizal colonization of *Cinnamomum camphora* seedlings grown with non-salt spray (CK), low-salt spray (LS), and high-salt spray (HS). Gt, Fm, and Gt+Fm represent three mycorrhizal treatments: inoculation with *Glomus tortuosum* and *Funneliformis mosseae*, alone and in combination, respectively. Box plots depict median values of plants, with points and bars indicating the variation between replicate plants and standard deviation, respectively (*n* = 3). Different lowercase letters indicate significant differences at *p* < 0.05 according to LSD.

In this study, mycorrhizal colonization was not observed in the roots of non-mycorrhizal *C. camphora* seedlings. All AM plants were infected with AMF, with root colonization ranging from 28% to 58% (Figure 1). Salt spray affected the root colonization of AM plants differently. The mycorrhizal colonization of *F. mosseae*- or combined fungi-inoculated plants significantly increased with increasing levels of salt spray. However, this trend was not observed in *G. tortuosum*-inoculated plants. Under non-saline conditions, root colonization did not differ significantly among AM plants. Under both low-salinity (LS) and high-salinity (HS) conditions, the root colonization of *F. mosseae*- or combined fungi-inoculated plants was higher than that of *G. tortuosum*-inoculated plants. Significant interactions between salt spray and AMF were observed for mycorrhizal colonization (Table S1).

### 3.2. Root Morphological Traits

**Figure 2:**
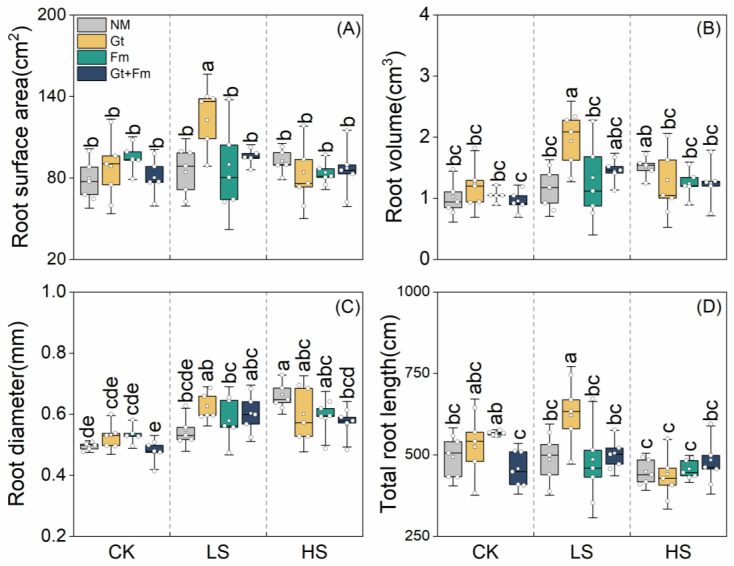
Effects of arbuscular mycorrhizal fungi on root surface area (**A**), root volume (**B**), root diameter (**C**), and total root length (**D**) of *Cinnamomum camphora* seedlings grown with non-salt spray (CK), low-salt spray (LS), and high-salt spray (HS). NM, Gt, Fm, and Gt+Fm represent four mycorrhizal treatments: inoculation with sterilized mycorrhizal fungi, with *Glomus tortuosum* and *Funneliformis mosseae*, alone and in combination, respectively. Box plots depict median values of plants, with points and bars indicating the variation between replicate plants and standard deviation, respectively (*n* = 5). Different lowercase letters indicate a significant difference at *p* < 0.05 according to LSD.

Neither salt spray nor AMF had any effect on root surface area (Figure 2A). Salt spray had no influence on the root volumes of plants, except for those inoculated with *G. tortuosum* (Figure 2B). AMF inoculation also had no effect on plant root volume. Salt spray significantly increased root diameter, whereas mycorrhizal colonization had no impact on it (Figure 2C). Salt spray notably decreased the total root length of plants, whereas AMF inoculation did not affect those of plants under control and HS conditions (Figure 2D). However, under LS conditions, especially with *G. tortuosum*, AMF significantly enhanced root length. Significant interactions between salt spray and AMF were observed for both the root diameter and total root length (Table S1).

### 3.3. Elemental Concentrations and Their Ratios

**Figure 3:**
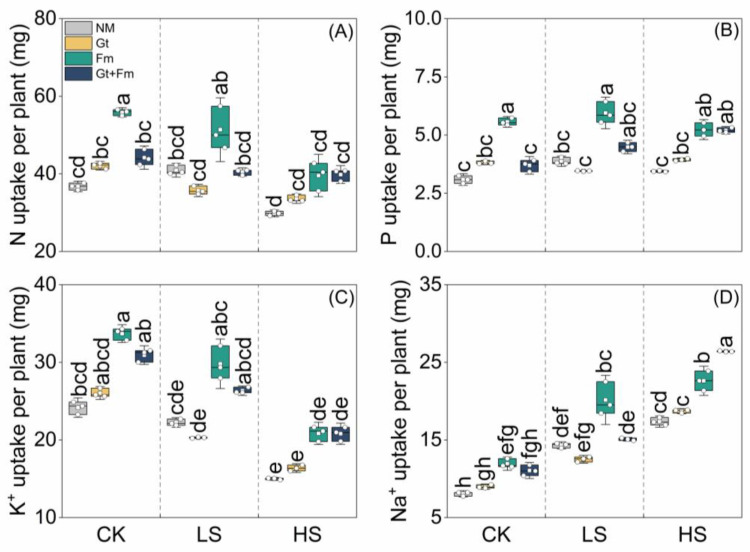
Effects of arbuscular mycorrhizal fungi on the uptake of N (**A**), P (**B**), K^+^ (**C**), and Na^+^ (**D**) of *Cinnamomum camphora* seedlings grown at non-salt spray (CK), low-salt spray (LS), and high-salt spray (HS). NM, Gt, Fm, and Gt+Fm represent four mycorrhizal treatments: inoculation with sterilized mycorrhizal fungi, with *Glomus tortuosum* and *Funneliformis mosseae*, alone and in combination, respectively. Box plots depict median values of plants, with points and bars indicating the variation between replicate plants and standard deviation, respectively (*n* = 3). Different lowercase letters indicate a significant difference at *p* < 0.05 according to LSD.

Salt spray had no effect on the N uptake of either NM plants or *G. tortuosum*-inoculated plants. However, it significantly decreased the N uptake of *F. mosseae*- or combined fungi-inoculated plants at high salt spray levels by 7.6% and 8.3%, respectively, compared to that of NM control plants (Figure 3A). Mycorrhizal inoculation, particularly with *F. mosseae*, notably increased N uptake in plants not exposed to salt spray. However, it had no effect on plants subjected to LS and HS. Except for combined fungi-inoculated plants, salt spray had no effect on the P uptake of all plants (Figure 3B). Inoculation with AMF, particularly with *F. mosseae*, significantly enhanced P uptake in plants under non-saline and LS conditions. Under HS conditions, both *F. mosseae* and combined fungi greatly improved P uptake, with no significant differences between them. High salt spray significantly decreased K^+^ uptake in NM, *G. tortuosum*-inoculated, *F. mosseae*-inoculated, and combined fungi-inoculated plants by 37.9%, 32.3%, 13.8%, and 13.9%, respectively, compared to NM control plants (Figure 3C). There were no differences observed between NM and AM plants with the exception of *F. mosseae*-inoculated plants in non-saline conditions. The Na^+^ uptake of all plants greatly increased with increasing salt spray levels. Under HS conditions, the Na^+^ uptake of NM, *G. tortuosum*-inoculated, *F. mosseae*-inoculated, and combined fungi-inoculated plants increased by 115.5%, 108.5%, 89.4%, and 138.7%, respectively, compared to their counterparts grown under non-saline conditions (Figure 3D). Mycorrhizal colonization had variable effects on Na^+^ uptake in plants at any salt spray level. 

**Figure 4:**
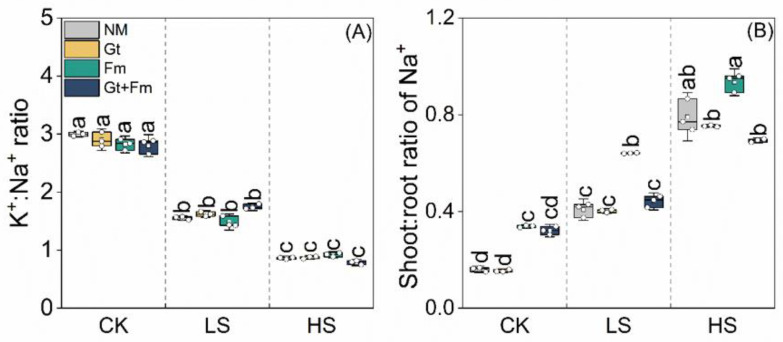
Effects of arbuscular mycorrhizal fungi on the K^+^:Na^+^ ratio (**A**) and shoot:root ratio of Na^+^ (**B**) of *Cinnamomum camphora* seedlings grown at non-salt spray (CK), low-salt spray (LS), and high-salt spray (HS). NM, Gt, Fm, and Gt+Fm represent four mycorrhizal treatments: inoculation with sterilized mycorrhizal fungi, with *Glomus tortuosum* and *Funneliformis mosseae*, alone and in combination, respectively. Box plots depict median values of plants, with points and bars indicating the variation between replicate plants and standard deviation, respectively (*n* = 3). Different lowercase letters indicate significant differences at *p* < 0.05 according to LSD.

Salt spray significantly decreased the K^+^/Na^+^ ratio in NM, *G. tortuosum*-inoculated, *F. mosseae*-inoculated, and combined fungi-inoculated plants under HS conditions by 71%, 71%, 69%, and 74%, respectively, compared to NM plants without salt spray (Figure 4A). The K^+^/Na^+^ ratio of NM and AM plants did not differ. Salt spray greatly increased the shoot/root ratio of Na^+^ in the plants (Figure 4B). Under HS conditions, the shoot/root ratios of Na^+^ in NM, *G. tortuosum*-inoculated, *F. mosseae*-inoculated, and combined fungi-inoculated plants increased by 392%, 388%, 176%, and 116%, respectively, compared to their counterparts grown under non-saline conditions. AMF had variable effects on the shoot/root ratio of Na^+^. Under HS conditions, the lowest value was observed in the combined fungi-inoculated plants.

**Figure 5:**
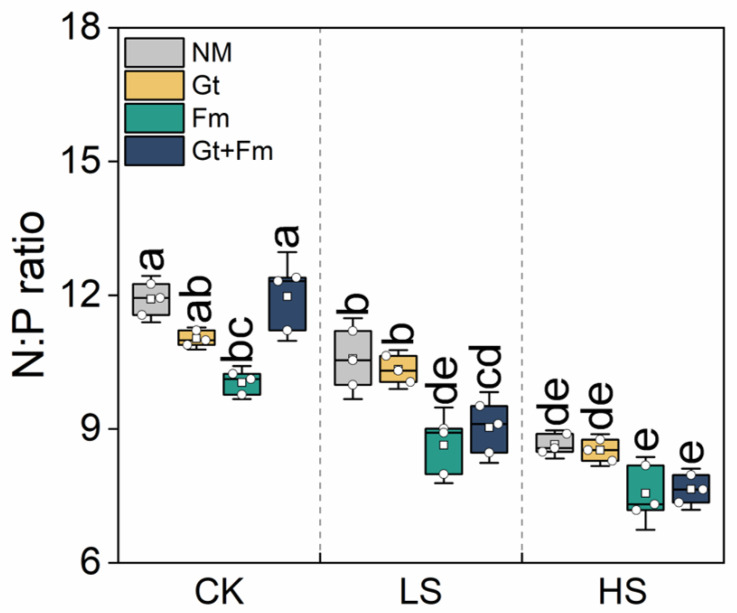
Effect of arbuscular mycorrhizal fungi on the N:P ratio of *Cinnamomum camphora* seedlings grown at non-salt spray (CK), low-salt spray (LS), and high-salt spray (HS). NM, Gt, Fm, and Gt+Fm represent four mycorrhizal treatments: inoculation with sterilized mycorrhizal fungi, with *Glomus tortuosum* and *Funneliformis mosseae*, alone and in combination, respectively. Box plots depict median values of plants, with points and bars indicating the variation between replicate plants and standard deviation, respectively (*n* = 3). Different lowercase letters indicate significant differences at *p* < 0.05 according to LSD.

Salt spray significantly decreased the N/P ratio, with average values ranging from 7.6 to 11.9 (Figure 5). Almost all N/P ratios of AM plants were lower than those of NM plants. Significant salt spray–AMF interactions were observed at the aforementioned elemental concentrations and ratios (Table S1).

### 3.4. Biomass Accumulation and Allocation

**Figure 6:**
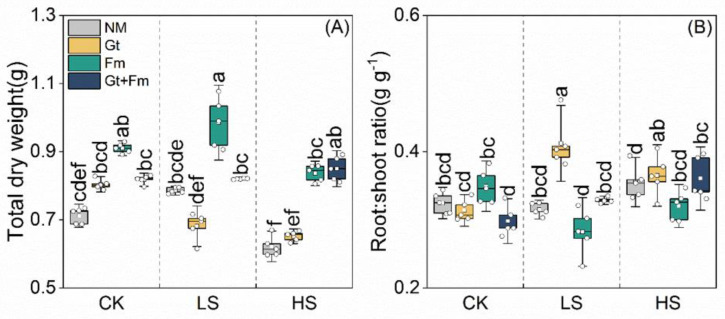
Effects of arbuscular mycorrhizal fungi on total dry weight (**A**) and root:shoot ratio (**B**) of *Cinnamomum camphora* seedlings grown at non-salt spray (CK), low-salt spray (LS), and high-salt spray (HS). NM, Gt, Fm, and Gt+Fm represent four mycorrhizal treatments: inoculation with sterilized mycorrhizal fungi, with *Glomus tortuosum* and *Funneliformis mosseae*, alone and in combination, respectively. Box plots depict median values of plants, with points and bars indicating the variation between replicate plants and standard deviation, respectively (*n* = 5). Different lowercase letters indicate significant differences at *p* < 0.05 according to LSD.

Except for *F. mosseae*- and combined fungi-inoculated plants, salt spray significantly decreased the total dry weights of NM- and *G. tortuosum*-inoculated plants by 12.7% and 8.5%, respectively, when compared with their counterparts under non-saline conditions (Figure 6A). Regardless of the total dry weight of *G. tortuosum*-inoculated plants under LS conditions, the total dry weights of AM plants were consistently higher than those of NM plants at all salt spray levels. The highest values were obtained for *F. mosseae*-inoculated plants grown under non-saline and LS conditions and for combined fungi-inoculated plants grown under HS conditions. Salt spray had no effect on the root/shoot ratios of NM plants but had variable effects on AM plants (Figure 6B). The mycorrhizal efficiencies on the root/shoot ratios varied among AM fungal species. Significant interactions between salt spray and AMF were observed for both total dry weight and root/shoot ratio (Table S1).

### 3.5. Salinity Tolerance, Mycorrhizal Efficiencies, and Their Relationships with Other Plant Traits

Salt spray significantly reduced the ST values of NM and *F. mosseae*-inoculated plants but had no effect on those of *G. tortuosum*- and combined fungi-inoculated plants (Figure S1A). Under LS conditions, the ST values of NM plants were higher than those of AM plants, whereas the values of AM plants were higher than those of NM plants under HS conditions, regardless of *G. tortuosum* inoculation. With increasing salt spray concentration, the MGR values of AM plants first decreased and then increased (Figure S1B). When the plants were grown without salt spray, there were no differences in the MGR between AM fungal species, whereas the MGR values varied within AM fungal species for plants exposed to salt spray. Under the LS condition, the highest value was observed in *F. mosseae*-inoculated plants, whereas under the HS condition, the highest value was observed for combined fungi-inoculated plants. Significant interactions between salt spray and AMF were observed for ST and MGR (Table S1).

**Figure 7:**
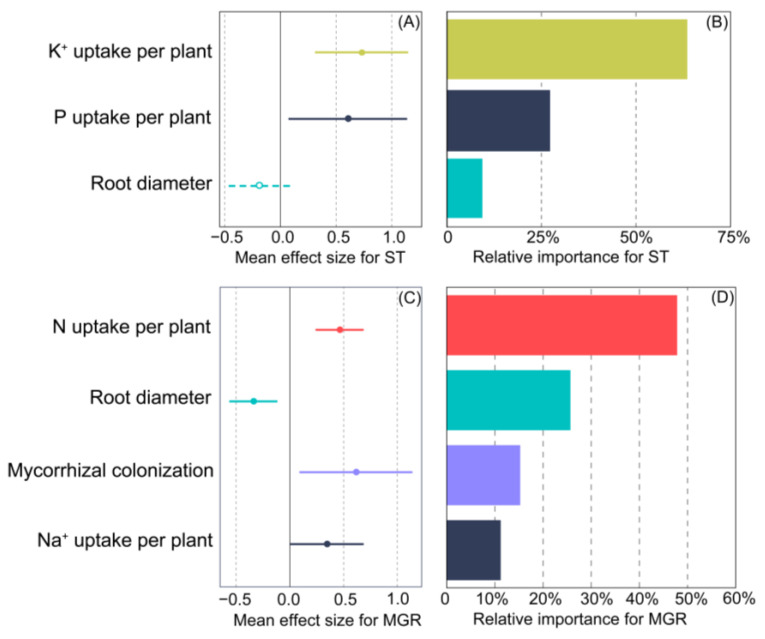
Mean effect sizes of observed variables on salinity tolerance (ST) (**A**) and mycorrhizal growth response (MGR) (**C**) and their relative contributions to ST (**B**) and MGR (**D**) in *Cinnamomum camphora* plants. Solid lines with filled circles demonstrate significant effects (*p* < 0.05), and the dotted lines with hollowed circles represent non-significant effects (*p* > 0.05).

ST was significantly and positively correlated with the uptake of K^+^ and P (Figure S2 and Figure 7A). MGR was significantly and positively correlated with mycorrhizal colonization and N uptake (Figure S2 and Figure 7C). To quantify the strength of their relationships with the determined plant parameters, the following models were constructed for ST (*R^2^* = 0.601, *p* < 0.000) and MGR (*R^2^* = 0.726, *p* < 0.000), respectively:

(3) $$ST=-0.1864X_{1}+0.7299X_{2}+0.6059X_{3}$$

where X_1_–X_3_ represent root diameter, K^+^ uptake, and P uptake, respectively.

(4) $$MGR=0.6148X_{1}-0.3400X_{2}+0.4628X_{3}+0.3437X_{4}$$

where X_1_–X_4_ represent mycorrhizal colonization, root diameter, N uptake, and Na^+^ uptake, respectively.

Furthermore, the uptake of K^+^ and P accounted for 90.6% of the total variance in ST, indicating their contribution to ST (Figure 7B). In contrast, mycorrhizal colonization, root diameter, and N uptake explained 88.8% of the total variance in MGR, suggesting their contribution to MGR (Figure 7D).

## 4. Discussion

This study aimed to unravel the principles underlying the effects of AMF on root functional traits and growth of *C. camphora* seedlings subjected to salt spray. As hypothesized, our data show that AMF can mitigate the detrimental effects of salt spray on root traits, nutrient acquisition, biomass accumulation, and salinity tolerance in *C. camphora* seedlings. Furthermore, most of the mycorrhizal efficiencies depend on the salt spray concentration and fungal taxa. Enhanced effects were observed under HS conditions, where inoculation with combined fungi was more beneficial for plant growth than inoculation with other fungi. Most importantly, our results suggest that ST is enhanced by an increased uptake of K^+^ and P.

### 4.1. Mycorrhizal Efficiency on Plant Biomass

In this study, a significant decrease in biomass was observed in NM plants of *C. camphora* in response to increased salt spray, which is consistent with the results observed for *Scirpus nodosus* [54], *Triplasis purpurea* [55], *Solidago nemoralis* [56], and *Commelina erecta* subsp. *maritime* [57]. However, other researchers have reported negligible biomass responses of *Scaevola sericea* [12,58] and *Spinifex sericeus* [3] or even positive responses of *Crambe maritime* [10] and *Myrica pensylvanica* [59] to salt spray. Notably, the effects of airborne salt depend on the plant species and salinity intensity, ranging from decreases in growth to relatively little or no effect (even a positive effect) on plants [57]. This contradictory response could be explained by a biphasic model that predicts that plants may experience a two-phase growth response involving water stress and the following ionic effects; the second phase may be responsible for the salt-specific response of biomass [17]. Mycorrhizal inoculation, particularly with combined fungi, greatly enhanced the plant biomass compared with that of NM plants at high salt spray concentrations (Figure 6A), suggesting that AMF can mitigate the detrimental effects of salt spray on plant growth. Compelling evidence suggests that AMF enhances the biomass of plants subjected to salinity stress [30,37]. The growth enhancement of plants subjected to salt spray could be achieved through nutrient input [3]. The data further corroborate that plant biomass is positively correlated with the uptake of N, P, and K^+^ (Figure S2). Generally, mycorrhizal benefits for plant growth are mainly ascribed to the mycorrhiza-mediated improvement in plant P [22,60]. However, the present study showed that mycorrhizal efficiency was mainly enhanced by N uptake (Figure 7D), which may be because *C. camphora* seedlings were exposed to N limitation during biomass production (Figure 5) [61].

### 4.2. Mycorrhizal Efficiency on Elementary Absorption

N and P are essential macronutrients that play important roles in improving resistance to salinity [37]. Our results show that the uptake of N and P in NM plants did not change with an increase in salt spray concentration (Figure 3A,B). Meanwhile, plant N/P ratios decreased with an increase in salt spray concentration (Figure 5), suggesting N limitation, compared with their natural values, ranging from 12 to 13 [61]. Notably, mycorrhizal inoculation did not mitigate the adverse effects of salt spray on N limitation, potentially because AMF requires more N than their hosts [62], and the N nutrients they access in the soil would be maintained for their own use before being provided to their hosts [63]. Furthermore, plants supplied with more N often have larger and more pliable leaves, lower epidermal hair densities, and thinner cuticles, making them more susceptible to injury from salt spray [3]. Our data showed that mycorrhizal inoculation did not enhance N to the same extent as P, suggesting a trade-off between the N and P availability in mycorrhizal plants.

In this study, the reduction in K^+^ and the increased response of Na^+^ to increasing salt spray resulted in a reduction in the K^+^/Na^+^ ratios (Figure 3C,D and Figure 4A). Similar responses have been reported previously [10,57,64] and are probably due to the ability of Na^+^ to compete with K^+^ at transporting sites or binding sites that are essential for various cellular functions [30]. Excess Na^+^ can cause various osmotic and metabolic issues in plants [65]. Therefore, the ability to enhance salinity resistance is mainly attributed to the reduced uptake, increased extrusion, and compartmentalization of Na^+^ in plant organs [66,67]. In our study, mycorrhizal inoculation marginally increased the K^+^ uptake but did not decrease Na^+^ uptake in plants at all salt spray concentrations, which is inconsistent with the results of previous studies showing that AMF-inoculated plants have lower Na^+^ contents [30,37]. However, combined fungi inoculation accelerated Na^+^ absorption and accumulation in roots (Figure 4B), representing a possible biomarker of salinity tolerance [20,58]. Shoots were the main entry point of Na^+^ when the plants were constantly exposed to salt spray, and greater Na^+^ translocation from the shoot to the root would reduce the ionic toxicity to the shoot, resulting in partial adaptation to salt spray stress [3].

### 4.3. Potential Mechanism of Salt Tolerance Associated with Mycorrhizal Inoculation

Salinity tolerance is a complex process characterized by changes at the physiological, biochemical, and genetic levels [68]. The results of this study demonstrate that salt spray significantly affects plant variables. However, some researchers have reported that these effects are less pronounced compared to those of soilborne salinity [10,12,58]. In contrast, the results of a few field studies showed that salt spray is more detrimental to barley than soil salinity [64]. Salt spray simultaneously affects the aerial parts of plants and soil salinity. Therefore, treated plants were simultaneously exposed to air- and soil-borne salinity, and the factors affecting plant resistance to salt spray stress could be disputed [5,58,69].

The results of numerous studies suggest that the mycorrhiza-mediated alleviation of salinity stress involves multiple mechanisms such as the enhancement of nutrient uptake; maintenance of ionic homeostasis; and biochemical, physiological, molecular, and ultrastructural changes [30,37]. However, the general principles of plant resistance to salt spray have not been established. Some researchers have suggested that nutrient acquisition [3] and variations in biochemical content [20] are the major factors affecting plant resistance to salt spray. Our data show that the enhanced ability of mycorrhizal plants to resist salt spray is due to an enhanced uptake of K^+^ and P (Figure 7). Furthermore, combined fungi are more effective at enhancing salinity tolerance than a single fungus due to functional complementarity among fungal species [70].

It is critical to note that the present study was conducted in a greenhouse, where plants may differ from field plants in terms of their sensitivity to salt spray. In the field, the magnitude of the effect of salt spray on plant shoots varies depending on numerous factors, including wind speed, topography, plant height, and distance from the shore [55]. Therefore, other plant variables at individual and molecular levels, in addition to root functional traits, must be analyzed in future studies to understand the mechanisms underlying the responses of mycorrhizal plants to salt spray stress.

## 5. Conclusions

Mycorrhizal inoculation, especially with the combination of *F. mosseae* and *G. tortuosum*, increases the mycorrhizal colonization, total dry weight, root/shoot ratio, P uptake, and salinity tolerance of plants grown under HS conditions. This enhancement improves the resistance of plants to salt spray. Furthermore, the results of the present study suggest that the ability of AM plants to resist salt spray depends on an enhanced uptake of K^+^ and P. These findings could provide both theoretical and practical guidance for cultivating *C. camphora* plants in coastal areas.

## Data Availability

Not applicable.

## Supplementary Materials

The following supporting information can be downloaded at: https://www.mdpi.com/article/10.3390/jof9100964/s1, Figure S1: Effects of arbuscular mycorrhizal fungi and salt spray on the salinity tolerance (ST) and mycorrhizal growth response (MGR) of *Cinnamomum camphora* seedlings; Figure S2: Relationships between the variables of *Cinnamomum camphora* plants; Table S1: *F* values of Two-way ANOVA for the effects of salt spray, arbuscular mycorrhizal fungi (AMF) and their interactive effects on the parameters of *Cinnamomum camphora* (L.) Presl.
