# Supplementary material for: Resistance of Mycorrhizal Cinnamomum camphora Seedlings to Salt Spray Depends on K+ and P Uptake
Source: J Fungi (Basel). 2023 Sep 26;9(10):964. doi: 10.3390/jof9100964 (PMC10607215; doi:10.3390/jof9100964)
Supplement: Supplementary file 1 [file jof-09-00964-s001.zip › 1. jof-2658943-supplementary-pending review.pdf]

Table S1. List of the novel species *Stenotrophomyces fumitolerans* WT5, the representatives of the family *Saccharomycetaceae* and outgroup taxa from families *Debaryomycetaceae, Phaffomycetaceae*, and *Pichiaceae* used for multi-locus phylogenetic analysis. Type strains were used for the analysis.

Strains	GenBank accession number for sequences				
	ITS	18S	26S	*TEF1*	*RPB2*
Barnettozyma populi CBS 8094	NR_153632	JQ698877	EF550277	EF552501	JQ698938
Candida glabrata CBS 138	AY046165	AY046237	U44808	AF402029	AF527898
Cyberlindnera americana CBS 5644	NR_152479	JQ698878	EF550328	EF552552	JQ698940
Debaryomyces hansenii JCM 1990	NR_120016	JQ698910	JQ689041	JQ699068	JQ698984
Dekkera bruxellensis CBS 72	NR_165974	JQ698898	JQ689028	JQ699055	JQ698969
Eremothecium cymbalariae NRRL Y-17582	AY046219	JQ698891	JQ689024	AF402090	JQ698962
Eremothecium gossypii NRRL Y-1056	AY046216	AY046265	U43389	AF402087	AY497595
Kazachstania africana CBS 2517	AY046155	AY046229	AY048159	AF402019	AF527887
Kazachstania exigua NRRL Y-12640	AY046170	X98868	AY048163	AF402034	AF527902
Kazachstania humilis CBS 5658	AY046174	AY046243	U69878	AF402039	AF527906
Kazachstania lodderae CBS 2757	AY046160	X83824	AY048161	AF402024	AF527893
Kazachstania martiniae CBS 6334	AY046157	AY046231	AF398481	AF402021	AF527890
Kazachstania piceae CBS 7738	AY046159	AY046233	U84346	AF402023	AF527892
Kazachstania servazzii CBS 4311	AY046153	Z75581	AY048157	AF402017	AF527885
Kazachstania telluris CBS 2685	AY046164	AY046236	U72158	AF402028	AF527897
Kazachstania viticola CBS 6463	AY046162	JQ698889	JQ689020	AF402026	AF527895
Kluyveromyces lactis NRRL Y-8278	AY046213	AY046264	U94919	AF402084	AY497620
Kluyveromyces marxianus NRRL Y-8281	AY046214	X89523	JQ689023	AF402085	JQ698961
Kregervanrija fluxuum CBS 2287	NR_111196	JQ698897	EF550268	JQ699054	JQ698968
Lachancea kluyveri CBS 3082	AY046209	Z75580	U68552	AF402079	AY497609
Lachancea thermotolerans CBS 6340	AY046207	X89526	JQ689022	AF402077	JQ698960
Lodderomyces elongisporus CBS 2605	NR_111593	JQ698906	JQ689035	JQ699062	JQ698978
Meyerozyma guilliermondii CBS 2030	NR_111247	JQ698913	JQ689047	JQ699074	JQ698990
Millerozyma farinosa CBS 185	NR_111254	AB054281	JQ689046	JQ699073	JQ698989
Nakaseomyces delphensis CBS 2170	AY046166	X83823	JQ689014	AF402030	AF527899
Naumovozyma castellii CBS 4309	AY046180	Z75577	AY048167	AF402045	AY548498
Naumovozyma dairenensis CBS 421	AY046181	Z75579	JQ689019	AF402046	AF527908
Pichia membranifaciens CBS 107	NR_111195	JQ698896	EF550227	JQ699053	JQ698967
Saccharomyces cerevisiae NRRL Y-12632	AY046146	EU011664	JQ689017	AF402004	AF527884
Saccharomyces kudriavzevii CBS 8840	AY046150	AY046226	AF398480	AF402011	KC881091
Saccharomycodes ludwigii NRRL Y-12793	AY046204	JQ698892	U73601	AF402074	JQ698963
Saturnispora dispora CBS 794	NR_155832	JQ698895	JQ689027	JQ699052	JQ698966
Schizosaccharomyces pombe ATCC 38366	NR121563.1	AY046272	JQ689077	AF402093	NM_001018498
Starmera amethionina CBS 6940	CBS data	JQ698876	JQ689006	EF552506	JQ698937
Stenotrophomyces fumitolerans WT5	OQ708375	OQ708375	OQ708375	OR661267	OQ715316
Tetrapisispora arboricola CBS 8765	NR_155927	NG_064806	NG_058411	AF402043	GCA_030557565
Tetrapisispora blattae CBS 6284	NR_155928	NG_063244	KY109818	AF402040	GCF_000315915
Tetrapisispora phaffii CBS 4417	AY046176	JQ698887	JQ689013	AF402041	JQ698951
Torulaspora delbrueckii NRRL Y-866	AY046187	X98120	JQ689018	AF402052	JQ698956
Vanderwaltozyma polyspora CBS 2163	AY046182	JQ698890	JQ689021	AF402047	AF527909
Wickerhamomyces anomalus NRRL Y-366	AY046221	EF550479	U74592	AF402092	EF599479
Wickerhamomyces canadensis CBS 1992	KY105899	EF550438	JQ689007	EF552524	JQ698939
Yueomyces sinensis CBS 7075	NR_159012	KP866235	NG_055015	KP866224	KP866228

Zygosaccharomyces bailii CBS 680	AY046191	X91083	U72161	AF402060	KC678068
Zygotorulaspora dagestanica KBP:Y-4591	MW036827	MW036827	MW036827	LR884242	LR884244
Zygotorulaspora florentina CBS 746	AY046168	X91086	U72165	AF402032	AF527900
Zygosaccharomyces kombuchaensis CBS 8849	AY046193	AF339890	AF339904	AF402062	KT728704
Zygotorulaspora mrakii CBS 4218	AY046169	JQ698888	JQ689015	AF402033	AF527901
Zygosaccharomyces rouxii CBS 732	AY046189	AY046251	JQ689016	AF402054	JQ698954
