# Supplementary figures and images for: Resistance of Mycorrhizal Cinnamomum camphora Seedlings to Salt Spray Depends on K+ and P Uptake
Source: J Fungi (Basel). 2023 Sep 26;9(10):964. doi: 10.3390/jof9100964 (PMC10607215; doi:10.3390/jof9100964)

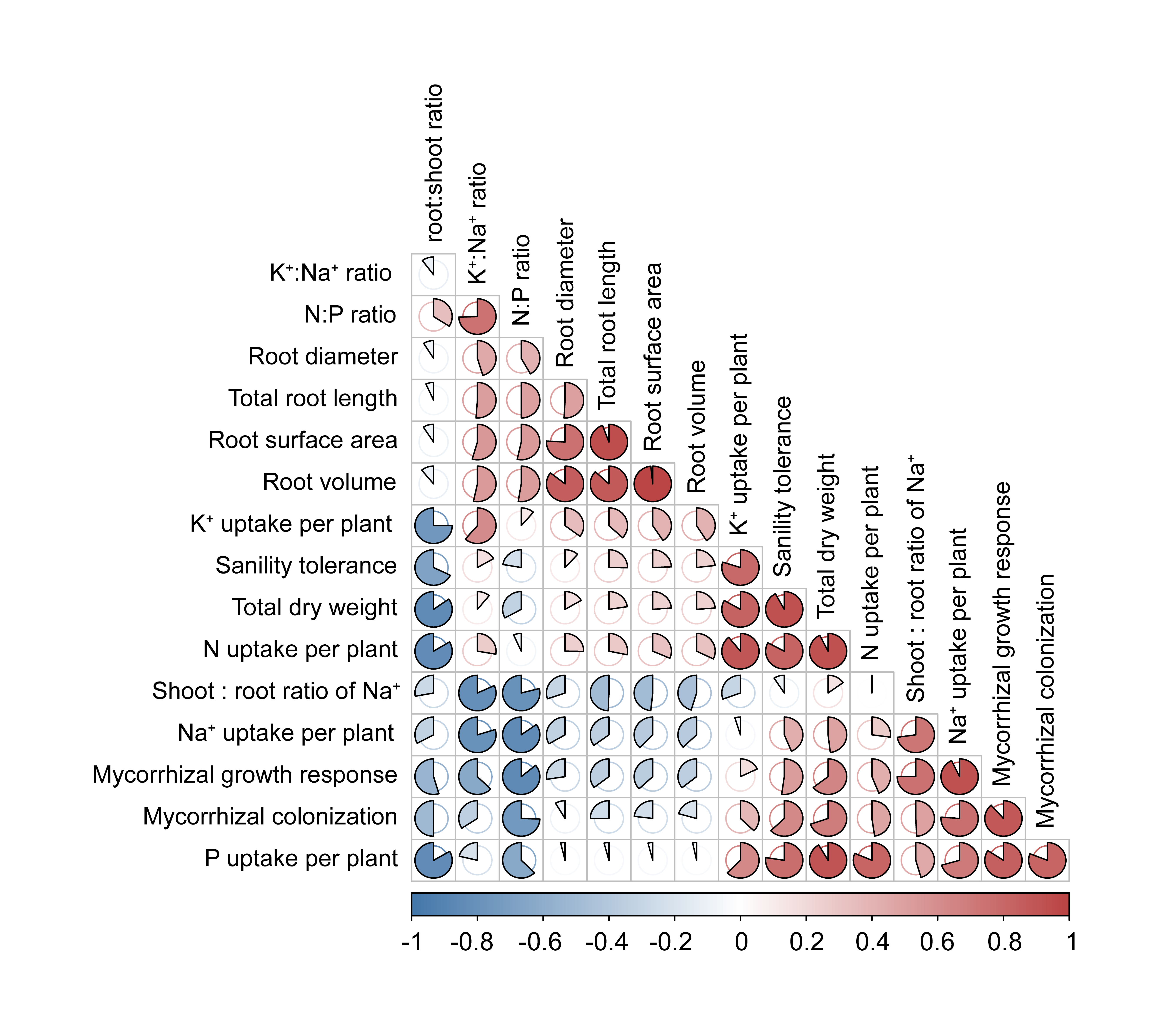

Supplement: Supplementary file 1 [file jof-09-00964-s001.zip › Figure S2.tif]
